# Supplementary material for: Multi‐dataset identification of innovative feature genes and molecular mechanisms in keratoconus
Source: J Cell Mol Med. 2024 Sep 19;28(18):e70079. doi: 10.1111/jcmm.70079 (PMC11412914; doi:10.1111/jcmm.70079)
Supplement: Supplementary file 2 — Material S2. [file JCMM-28-e70079-s002.zip › 7.GSEA analysis/3.DBNDD1/KEGG_MAPK_SIGNALING_PATHWAY.html]

Details for gene set KEGG\_MAPK\_SIGNALING\_PATHWAY[GSEA]

|  || Dataset | DBNDD1.group.cls#h\_versus\_l |
| Phenotype | group.cls#h\_versus\_l |
| Upregulated in class | l |
| GeneSet | KEGG\_MAPK\_SIGNALING\_PATHWAY |
| Enrichment Score (ES) | -0.29015377 |
| Normalized Enrichment Score (NES) | -1.209231 |
| Nominal p-value | 0.1754386 |
| FDR q-value | 0.691389 |
| FWER p-Value | 0.988 |
Table: GSEA Results Summary

  

Fig 1: Enrichment plot: KEGG\_MAPK\_SIGNALING\_PATHWAY      
 Profile of the Running ES Score & Positions of GeneSet Members on the Rank Ordered List

  

| SYMBOL | TITLE | RANK IN GENE LIST | RANK METRIC SCORE | RUNNING ES | CORE ENRICHMENT || 1 | FGF16 | na | 48 | 0.676 | 0.0214 | No |
| 2 | CACNB1 | na | 587 | 0.431 | 0.0203 | No |
| 3 | FGF14 | na | 999 | 0.383 | 0.0213 | No |
| 4 | PPP3R2 | na | 1210 | 0.361 | 0.0273 | No |
| 5 | CACNA1I | na | 1237 | 0.359 | 0.0387 | No |
| 6 | CACNA1F | na | 1766 | 0.316 | 0.0340 | No |
| 7 | RAC3 | na | 1824 | 0.312 | 0.0428 | No |
| 8 | MECOM | na | 1978 | 0.301 | 0.0485 | No |
| 9 | FGF8 | na | 2224 | 0.286 | 0.0510 | No |
| 10 | FGF17 | na | 2525 | 0.271 | 0.0515 | No |
| 11 | CACNG5 | na | 2873 | 0.257 | 0.0500 | No |
| 12 | FGF23 | na | 3173 | 0.245 | 0.0496 | No |
| 13 | PDGFRA | na | 3213 | 0.243 | 0.0567 | No |
| 14 | ELK1 | na | 3923 | 0.216 | 0.0434 | No |
| 15 | PTPN5 | na | 4058 | 0.212 | 0.0466 | No |
| 16 | CACNG7 | na | 4144 | 0.209 | 0.0512 | No |
| 17 | CACNA1B | na | 4164 | 0.208 | 0.0576 | No |
| 18 | CACNA1H | na | 4227 | 0.205 | 0.0628 | No |
| 19 | TGFBR2 | na | 4515 | 0.197 | 0.0610 | No |
| 20 | CACNA1A | na | 5029 | 0.180 | 0.0522 | No |
| 21 | FGF21 | na | 5114 | 0.176 | 0.0557 | No |
| 22 | ARRB1 | na | 5893 | 0.153 | 0.0383 | No |
| 23 | HSPA2 | na | 5905 | 0.153 | 0.0431 | No |
| 24 | PRKCG | na | 6020 | 0.150 | 0.0449 | No |
| 25 | NFATC2 | na | 6248 | 0.144 | 0.0431 | No |
| 26 | FGF18 | na | 6694 | 0.132 | 0.0347 | No |
| 27 | PLA2G2F | na | 6705 | 0.131 | 0.0388 | No |
| 28 | RASGRP2 | na | 6889 | 0.127 | 0.0377 | No |
| 29 | PLA2G10 | na | 7003 | 0.124 | 0.0386 | No |
| 30 | RPS6KA2 | na | 7249 | 0.119 | 0.0355 | No |
| 31 | FLNB | na | 7307 | 0.118 | 0.0379 | No |
| 32 | HSPA8 | na | 7790 | 0.108 | 0.0275 | No |
| 33 | HSPA1L | na | 7968 | 0.104 | 0.0259 | No |
| 34 | DUSP9 | na | 7983 | 0.104 | 0.0290 | No |
| 35 | PLA2G2D | na | 8134 | 0.101 | 0.0280 | No |
| 36 | CACNG1 | na | 8316 | 0.098 | 0.0261 | No |
| 37 | SOS1 | na | 8586 | 0.093 | 0.0214 | No |
| 38 | FGF9 | na | 8610 | 0.092 | 0.0238 | No |
| 39 | CHP2 | na | 8667 | 0.091 | 0.0253 | No |
| 40 | FASLG | na | 8688 | 0.091 | 0.0278 | No |
| 41 | FGF12 | na | 8755 | 0.090 | 0.0289 | No |
| 42 | TAOK2 | na | 8814 | 0.089 | 0.0302 | No |
| 43 | PLA2G12B | na | 9010 | 0.085 | 0.0274 | No |
| 44 | PDGFA | na | 9340 | 0.081 | 0.0206 | No |
| 45 | AKT2 | na | 9388 | 0.080 | 0.0219 | No |
| 46 | MAPK3 | na | 9865 | 0.073 | 0.0106 | No |
| 47 | PDGFRB | na | 10046 | 0.071 | 0.0077 | No |
| 48 | NTRK1 | na | 10107 | 0.070 | 0.0083 | No |
| 49 | CACNA2D3 | na | 10208 | 0.068 | 0.0077 | No |
| 50 | RASGRF1 | na | 10469 | 0.065 | 0.0024 | No |
| 51 | MAPK10 | na | 10497 | 0.065 | 0.0038 | No |
| 52 | MAPK11 | na | 10512 | 0.064 | 0.0055 | No |
| 53 | MAP2K5 | na | 10752 | 0.061 | 0.0007 | No |
| 54 | MAP2K2 | na | 10931 | 0.059 | -0.0025 | No |
| 55 | MAPKAPK3 | na | 11253 | 0.055 | -0.0100 | No |
| 56 | FGF5 | na | 11314 | 0.054 | -0.0099 | No |
| 57 | MAPK9 | na | 11537 | 0.052 | -0.0146 | No |
| 58 | MAPK13 | na | 11811 | 0.049 | -0.0208 | No |
| 59 | FGFR4 | na | 11906 | 0.048 | -0.0220 | No |
| 60 | DUSP3 | na | 12010 | 0.047 | -0.0234 | No |
| 61 | FGF22 | na | 12142 | 0.046 | -0.0256 | No |
| 62 | FGFR3 | na | 12198 | 0.045 | -0.0257 | No |
| 63 | RASA1 | na | 12494 | 0.043 | -0.0328 | No |
| 64 | MAX | na | 12590 | 0.042 | -0.0342 | No |
| 65 | MKNK2 | na | 12747 | 0.040 | -0.0373 | No |
| 66 | MAP3K6 | na | 12941 | 0.039 | -0.0416 | No |
| 67 | FGF2 | na | 13023 | 0.038 | -0.0427 | No |
| 68 | HSPA1A | na | 13025 | 0.038 | -0.0415 | No |
| 69 | NF1 | na | 13175 | 0.037 | -0.0445 | No |
| 70 | CACNA1D | na | 13311 | 0.036 | -0.0473 | No |
| 71 | CASP3 | na | 13339 | 0.035 | -0.0468 | No |
| 72 | FOS | na | 13400 | 0.035 | -0.0474 | No |
| 73 | MKNK1 | na | 13544 | 0.034 | -0.0504 | No |
| 74 | SOS2 | na | 13580 | 0.033 | -0.0503 | No |
| 75 | TGFB3 | na | 13624 | 0.033 | -0.0505 | No |
| 76 | MAP3K3 | na | 13743 | 0.032 | -0.0528 | No |
| 77 | TP53 | na | 13962 | 0.030 | -0.0581 | No |
| 78 | CACNB2 | na | 14012 | 0.030 | -0.0585 | No |
| 79 | PLA2G6 | na | 14048 | 0.030 | -0.0585 | No |
| 80 | KRAS | na | 14127 | 0.029 | -0.0598 | No |
| 81 | RASGRP4 | na | 14161 | 0.029 | -0.0598 | No |
| 82 | PPM1B | na | 14256 | 0.028 | -0.0616 | No |
| 83 | RPS6KA4 | na | 14344 | 0.028 | -0.0632 | No |
| 84 | FGF3 | na | 14453 | 0.027 | -0.0654 | No |
| 85 | CD14 | na | 14548 | 0.026 | -0.0673 | No |
| 86 | RAC1 | na | 14759 | 0.025 | -0.0725 | No |
| 87 | RPS6KA5 | na | 14808 | 0.024 | -0.0731 | No |
| 88 | NLK | na | 15329 | 0.020 | -0.0875 | No |
| 89 | CACNG8 | na | 15428 | 0.020 | -0.0897 | No |
| 90 | BRAF | na | 15471 | 0.019 | -0.0903 | No |
| 91 | TAOK1 | na | 15652 | 0.018 | -0.0949 | No |
| 92 | PLA2G4A | na | 15676 | 0.018 | -0.0949 | No |
| 93 | FGF20 | na | 15746 | 0.018 | -0.0963 | No |
| 94 | PRKX | na | 15799 | 0.017 | -0.0973 | No |
| 95 | MAP3K4 | na | 16236 | 0.014 | -0.1094 | No |
| 96 | RPS6KA3 | na | 16816 | 0.011 | -0.1259 | No |
| 97 | RAP1A | na | 16884 | 0.010 | -0.1275 | No |
| 98 | TRAF6 | na | 17024 | 0.009 | -0.1312 | No |
| 99 | HSPB1 | na | 17168 | 0.008 | -0.1351 | No |
| 100 | STK3 | na | 17487 | 0.006 | -0.1441 | No |
| 101 | MAPK14 | na | 17505 | 0.006 | -0.1444 | No |
| 102 | PLA2G2E | na | 17656 | 0.005 | -0.1486 | No |
| 103 | MAPK7 | na | 17658 | 0.005 | -0.1484 | No |
| 104 | FGF10 | na | 17845 | 0.004 | -0.1537 | No |
| 105 | FGF19 | na | 17912 | 0.003 | -0.1555 | No |
| 106 | RAP1B | na | 18018 | 0.003 | -0.1584 | No |
| 107 | FGF7 | na | 18036 | 0.002 | -0.1588 | No |
| 108 | MAP3K1 | na | 18103 | 0.002 | -0.1607 | No |
| 109 | PLA2G12A | na | 18106 | 0.002 | -0.1607 | No |
| 110 | TAB1 | na | 18110 | 0.002 | -0.1607 | No |
| 111 | MOS | na | 18122 | 0.002 | -0.1610 | No |
| 112 | FGF13 | na | 18153 | 0.002 | -0.1618 | No |
| 113 | HSPA1B | na | 18247 | 0.001 | -0.1644 | No |
| 114 | PPP3R1 | na | 18349 | 0.000 | -0.1674 | No |
| 115 | STK4 | na | 18367 | 0.000 | -0.1679 | No |
| 116 | MAP3K11 | na | 18513 | -0.001 | -0.1720 | No |
| 117 | PPP5C | na | 18539 | -0.001 | -0.1727 | No |
| 118 | MAP2K4 | na | 18667 | -0.002 | -0.1763 | No |
| 119 | ARRB2 | na | 18679 | -0.002 | -0.1766 | No |
| 120 | DAXX | na | 18892 | -0.004 | -0.1826 | No |
| 121 | ELK4 | na | 18946 | -0.004 | -0.1840 | No |
| 122 | CHUK | na | 18953 | -0.004 | -0.1841 | No |
| 123 | HRAS | na | 18984 | -0.004 | -0.1848 | No |
| 124 | RAF1 | na | 19005 | -0.005 | -0.1852 | No |
| 125 | AKT1 | na | 19066 | -0.005 | -0.1868 | No |
| 126 | TGFB2 | na | 19303 | -0.007 | -0.1934 | No |
| 127 | EGF | na | 19333 | -0.007 | -0.1940 | No |
| 128 | PLA2G4E | na | 19468 | -0.008 | -0.1976 | No |
| 129 | FGF6 | na | 19523 | -0.009 | -0.1989 | No |
| 130 | RELB | na | 19734 | -0.011 | -0.2046 | No |
| 131 | MAP4K1 | na | 19766 | -0.011 | -0.2051 | No |
| 132 | MAP2K7 | na | 19930 | -0.012 | -0.2094 | No |
| 133 | ATF4 | na | 20029 | -0.013 | -0.2118 | No |
| 134 | FGF11 | na | 20046 | -0.013 | -0.2119 | No |
| 135 | FLNC | na | 20050 | -0.013 | -0.2115 | No |
| 136 | DUSP16 | na | 20253 | -0.015 | -0.2168 | No |
| 137 | TAOK3 | na | 20343 | -0.016 | -0.2189 | No |
| 138 | PTPRR | na | 20388 | -0.016 | -0.2196 | No |
| 139 | GNA12 | na | 20454 | -0.017 | -0.2209 | No |
| 140 | PRKACA | na | 20814 | -0.020 | -0.2307 | No |
| 141 | RAC2 | na | 20865 | -0.021 | -0.2314 | No |
| 142 | PRKCA | na | 21412 | -0.026 | -0.2464 | No |
| 143 | MAP3K13 | na | 21429 | -0.026 | -0.2460 | No |
| 144 | MAPKAPK2 | na | 21941 | -0.031 | -0.2597 | No |
| 145 | ECSIT | na | 21967 | -0.032 | -0.2594 | No |
| 146 | EGFR | na | 22041 | -0.033 | -0.2604 | No |
| 147 | TRAF2 | na | 22056 | -0.033 | -0.2597 | No |
| 148 | MAPK8IP1 | na | 22063 | -0.033 | -0.2588 | No |
| 149 | GRB2 | na | 22294 | -0.035 | -0.2643 | No |
| 150 | IKBKB | na | 22300 | -0.035 | -0.2633 | No |
| 151 | PLA2G2C | na | 22365 | -0.036 | -0.2639 | No |
| 152 | RPS6KA6 | na | 22401 | -0.036 | -0.2637 | No |
| 153 | RAPGEF2 | na | 22403 | -0.036 | -0.2625 | No |
| 154 | CHP1 | na | 22407 | -0.036 | -0.2614 | No |
| 155 | PPP3CA | na | 22457 | -0.037 | -0.2616 | No |
| 156 | MAPT | na | 22472 | -0.037 | -0.2607 | No |
| 157 | MAP2K3 | na | 22519 | -0.037 | -0.2608 | No |
| 158 | MAPK8 | na | 22620 | -0.039 | -0.2624 | No |
| 159 | CDC25B | na | 22622 | -0.039 | -0.2611 | No |
| 160 | CDC42 | na | 22626 | -0.039 | -0.2599 | No |
| 161 | MYC | na | 22660 | -0.039 | -0.2595 | No |
| 162 | MAPK1 | na | 22705 | -0.040 | -0.2595 | No |
| 163 | TNFRSF1A | na | 22847 | -0.041 | -0.2622 | No |
| 164 | PPP3CB | na | 22975 | -0.043 | -0.2644 | No |
| 165 | MAP3K7 | na | 23011 | -0.043 | -0.2640 | No |
| 166 | MAPKAPK5 | na | 23028 | -0.043 | -0.2630 | No |
| 167 | DUSP14 | na | 23033 | -0.043 | -0.2617 | No |
| 168 | IKBKG | na | 23063 | -0.044 | -0.2610 | No |
| 169 | JMJD7-PLA2G4B | na | 23109 | -0.044 | -0.2608 | No |
| 170 | CACNA1C | na | 23124 | -0.045 | -0.2597 | No |
| 171 | CRK | na | 23147 | -0.045 | -0.2589 | No |
| 172 | MAP4K4 | na | 23177 | -0.045 | -0.2582 | No |
| 173 | RRAS | na | 23237 | -0.046 | -0.2583 | No |
| 174 | FGF1 | na | 23262 | -0.046 | -0.2575 | No |
| 175 | DUSP8 | na | 23934 | -0.055 | -0.2751 | No |
| 176 | JUN | na | 24077 | -0.057 | -0.2773 | No |
| 177 | LAMTOR3 | na | 24215 | -0.059 | -0.2792 | No |
| 178 | NTF3 | na | 24248 | -0.060 | -0.2782 | No |
| 179 | DUSP6 | na | 24295 | -0.060 | -0.2775 | No |
| 180 | PAK2 | na | 24360 | -0.061 | -0.2773 | No |
| 181 | MAPK8IP3 | na | 24443 | -0.062 | -0.2775 | No |
| 182 | FAS | na | 24662 | -0.066 | -0.2816 | No |
| 183 | DUSP1 | na | 24664 | -0.066 | -0.2795 | No |
| 184 | NFATC4 | na | 24826 | -0.068 | -0.2818 | No |
| 185 | RRAS2 | na | 24862 | -0.069 | -0.2805 | No |
| 186 | GNG12 | na | 24895 | -0.069 | -0.2791 | No |
| 187 | FGFR2 | na | 25276 | -0.075 | -0.2876 | Yes |
| 188 | DUSP7 | na | 25305 | -0.075 | -0.2859 | Yes |
| 189 | NTF4 | na | 25414 | -0.077 | -0.2864 | Yes |
| 190 | TAB2 | na | 25426 | -0.077 | -0.2842 | Yes |
| 191 | MAP3K2 | na | 25599 | -0.080 | -0.2864 | Yes |
| 192 | MAPK12 | na | 25666 | -0.081 | -0.2856 | Yes |
| 193 | RPS6KA1 | na | 25675 | -0.081 | -0.2831 | Yes |
| 194 | STMN1 | na | 25718 | -0.082 | -0.2816 | Yes |
| 195 | PPM1A | na | 25744 | -0.083 | -0.2795 | Yes |
| 196 | MAP4K2 | na | 25746 | -0.083 | -0.2767 | Yes |
| 197 | NFKB1 | na | 25842 | -0.084 | -0.2767 | Yes |
| 198 | MAPK8IP2 | na | 25887 | -0.085 | -0.2751 | Yes |
| 199 | GADD45G | na | 26045 | -0.088 | -0.2766 | Yes |
| 200 | RASGRF2 | na | 26133 | -0.090 | -0.2761 | Yes |
| 201 | JUND | na | 26161 | -0.091 | -0.2738 | Yes |
| 202 | TGFBR1 | na | 26390 | -0.095 | -0.2772 | Yes |
| 203 | HSPA6 | na | 26391 | -0.095 | -0.2740 | Yes |
| 204 | NGF | na | 26508 | -0.098 | -0.2741 | Yes |
| 205 | CACNA1G | na | 26511 | -0.098 | -0.2709 | Yes |
| 206 | MAP3K5 | na | 26663 | -0.101 | -0.2718 | Yes |
| 207 | PRKACB | na | 26730 | -0.102 | -0.2703 | Yes |
| 208 | PPP3CC | na | 26734 | -0.102 | -0.2670 | Yes |
| 209 | NFKB2 | na | 26748 | -0.102 | -0.2639 | Yes |
| 210 | NR4A1 | na | 26839 | -0.104 | -0.2630 | Yes |
| 211 | CRKL | na | 27010 | -0.108 | -0.2643 | Yes |
| 212 | NRAS | na | 27014 | -0.108 | -0.2608 | Yes |
| 213 | TNF | na | 27164 | -0.111 | -0.2613 | Yes |
| 214 | ATF2 | na | 27172 | -0.112 | -0.2578 | Yes |
| 215 | PAK1 | na | 28053 | -0.132 | -0.2788 | Yes |
| 216 | PRKACG | na | 28125 | -0.134 | -0.2764 | Yes |
| 217 | MAP4K3 | na | 28152 | -0.134 | -0.2726 | Yes |
| 218 | RASA2 | na | 28173 | -0.135 | -0.2687 | Yes |
| 219 | BDNF | na | 28409 | -0.141 | -0.2707 | Yes |
| 220 | CACNG4 | na | 28414 | -0.141 | -0.2661 | Yes |
| 221 | GADD45A | na | 28540 | -0.145 | -0.2648 | Yes |
| 222 | PLA2G3 | na | 28607 | -0.146 | -0.2618 | Yes |
| 223 | RELA | na | 28616 | -0.147 | -0.2571 | Yes |
| 224 | IL1B | na | 28762 | -0.150 | -0.2563 | Yes |
| 225 | PLA2G5 | na | 29074 | -0.159 | -0.2599 | Yes |
| 226 | SRF | na | 29148 | -0.161 | -0.2566 | Yes |
| 227 | MAP2K1 | na | 29246 | -0.164 | -0.2539 | Yes |
| 228 | CACNB3 | na | 29250 | -0.164 | -0.2485 | Yes |
| 229 | CACNA2D4 | na | 29251 | -0.164 | -0.2429 | Yes |
| 230 | MAP2K6 | na | 29536 | -0.173 | -0.2453 | Yes |
| 231 | DUSP4 | na | 29549 | -0.174 | -0.2398 | Yes |
| 232 | DUSP2 | na | 29940 | -0.187 | -0.2448 | Yes |
| 233 | FLNA | na | 30291 | -0.197 | -0.2484 | Yes |
| 234 | PTPN7 | na | 30489 | -0.203 | -0.2472 | Yes |
| 235 | IL1R2 | na | 30516 | -0.204 | -0.2411 | Yes |
| 236 | GADD45B | na | 30544 | -0.205 | -0.2350 | Yes |
| 237 | DDIT3 | na | 30704 | -0.211 | -0.2325 | Yes |
| 238 | TGFB1 | na | 30914 | -0.218 | -0.2312 | Yes |
| 239 | IL1A | na | 31036 | -0.222 | -0.2273 | Yes |
| 240 | CACNG6 | na | 31154 | -0.226 | -0.2230 | Yes |
| 241 | MAP3K8 | na | 31607 | -0.245 | -0.2279 | Yes |
| 242 | MEF2C | na | 31834 | -0.255 | -0.2259 | Yes |
| 243 | PLA2G1B | na | 31847 | -0.256 | -0.2176 | Yes |
| 244 | DUSP5 | na | 32028 | -0.265 | -0.2139 | Yes |
| 245 | MAP3K14 | na | 32153 | -0.273 | -0.2083 | Yes |
| 246 | NTRK2 | na | 32264 | -0.280 | -0.2021 | Yes |
| 247 | CACNA2D2 | na | 32648 | -0.304 | -0.2029 | Yes |
| 248 | RASGRP1 | na | 32709 | -0.307 | -0.1943 | Yes |
| 249 | MAP3K12 | na | 33137 | -0.340 | -0.1953 | Yes |
| 250 | CACNG2 | na | 33176 | -0.343 | -0.1848 | Yes |
| 251 | IL1R1 | na | 33185 | -0.343 | -0.1735 | Yes |
| 252 | AKT3 | na | 33656 | -0.385 | -0.1742 | Yes |
| 253 | MRAS | na | 33756 | -0.398 | -0.1637 | Yes |
| 254 | CACNA2D1 | na | 33832 | -0.408 | -0.1521 | Yes |
| 255 | FGFR1 | na | 33845 | -0.409 | -0.1387 | Yes |
| 256 | CACNB4 | na | 33927 | -0.420 | -0.1269 | Yes |
| 257 | DUSP10 | na | 34007 | -0.431 | -0.1147 | Yes |
| 258 | PDGFB | na | 34011 | -0.431 | -0.1002 | Yes |
| 259 | PRKCB | na | 34153 | -0.452 | -0.0891 | Yes |
| 260 | CACNA1S | na | 34256 | -0.474 | -0.0761 | Yes |
| 261 | CACNG3 | na | 34262 | -0.475 | -0.0602 | Yes |
| 262 | CACNA1E | na | 34285 | -0.482 | -0.0446 | Yes |
| 263 | PLA2G2A | na | 34424 | -0.522 | -0.0310 | Yes |
| 264 | RASGRP3 | na | 34491 | -0.548 | -0.0145 | Yes |
| 265 | FGF4 | na | 34605 | -0.633 | 0.0036 | Yes |
Table: GSEA details [plain text format]

  

Fig 2: KEGG\_MAPK\_SIGNALING\_PATHWAY      
 Blue-Pink O' Gram in the Space of the Analyzed GeneSet

  

Fig 3: KEGG\_MAPK\_SIGNALING\_PATHWAY: Random ES distribution      
 Gene set null distribution of ES for **KEGG\_MAPK\_SIGNALING\_PATHWAY**
